# Notices, Reviews, Etc.

**Published:** 1860-07

**Authors:** 


### Notices, Reviews, Etc.

*Blackwood's Magazine*, and British Reviews. L. Scott & Co., 54 Gold st., New-York, continue to publish the following leading British Periodicals: 1. London Quarterly, *Conservative;* 2. Edinburgh Review, *Whig;* 3. North British Review, *Free Church;* 4. Westminster Review, *Liberal;* 5. Blackwood's Magazine, *Tory;* presenting the three great political parties of Great Britain, Whig, Tory, and Radical. But politics form only one feature of their character; as organs of the most profound writers on science, literature, morality, and religion, they stand, as they ever have stood, unrivaled in the world of letters, indispensable to the scholar and the professional man, while to intelligent readers of every class, they furnish a more correct and satisfactory record of the current literature of the day, throughout the world, than can be possibly obtained from any other source, and at a cost extraordinarily small—of ten dollars a year for the five; three dollars each, singly. Any subscriber sending us a check for ten dollars, payable to the order of Leonard Scott & Co., will receive for one year the five publications above named, and Hall's JOURAL OF HEALTH from January last. In cities and large towns, *Blackwood* and the Reviews are delivered free of postage; elsewhere, *Blackwood* is twenty-four cents a year, each Review, fourteen cents.

The *British and Foreign Medico-Chirurgical Review;* or, Quarterly Journal of Practical Medicine and Surgery. Republished by S. S. & W. Wood, 389 Broadway, New-York, at $3 a year, in advance; free of postage, is in its twenty-fifth volume, and is now, as it always has been, a standard and sterling medical quarterly, which every educated physician ought to patronize.

*Boston Medical and Surgical Journal*, $3 a year, is a monthly in its sixty-second volume; is edited with industry and ability, and is widely patronized.

The *Christian Review.* E. G. Robinson, editor. Published at $3 a year, by Sheldon & Co., 115 Nassau street, New-York. Is one of the ablest quarterlies in the Protestant Church, and a credit to the Baptist Church, of whose doctrine and polity it is the exponent; its reviews of new publications are always written discriminately, conscientiously, and after a full examination; a line of conduct which, as is to be regretted, too many so-called reviews fail to follow, to the deception and injury of their patrons.

The same enterprising publishers isssue monthly, at $1 a year, the *Mother's Journal*, edited by that excellent woman, Mrs. Caroline O. Hiscox. We wish it could be, as it well deserves, a family visitant to multitudes of homes.

The *Home Monthly.* Buffalo, N. Y.; $1.50 a year, edited by Mrs. Arey and Gildersleeve; has the very suggestive motto: "There is a power behind the school-room and the church." It is judiciously edited, and its selections are always good.

The *Happy Home.* Boston, monthly, $2 a year, C. Stone & Co. Is largely drawn upon by the press, a substantial evidence of its value.

The *Ladies' Home Magazine.* By T. S. Arthur & Co., 323 Walnut street, Philadelphia, Pa., $2 a year, with the editorial aid of Virginia F. Townsend. Will profit and instruct and refine and elevate any family which secures it monthly.

*Godey's Lady's Book*, $3 a year, Philadelphia. This is The Pictorial Monthly. The most elegant in the Union, and distances all competition. Now in its sixtieth volume. Its practical value has been increased of late by the contributions of Dr. J. S. Wilson, of Georgia, to the Health Department.

